# Motor Competence in Adolescents: Exploring Association with Physical Fitness

**DOI:** 10.3390/sports7070176

**Published:** 2019-07-20

**Authors:** Thórdís Gísladóttir, Monika Haga, Hermundur Sigmundsson

**Affiliations:** 1 Center for Health and Sport Science, School of Education, University of Iceland, 105 Reykjavik, Iceland; 2 Department of Teacher Education, Norwegian University of Science and Technology, 7491 Trondheim, Norway; 3 Department of Psychology, Norwegian University of Science and Technology, 7491 Trondheim, Norway; 4 Sport Science Department, Reykjavik University, 101 Reykjavik, Iceland

**Keywords:** motor skills, physical fitness, motor competence, adolescents

## Abstract

The purpose of this study was twofold: First, to examine the correlation between adolescents’ performance on the Movement Assessment Battery for Children -2 (MABC-2) and the Test of Motor Competence (TMC), and second, to interpret the correlation between performance on physical fitness measures and motor competence. This study had a cross-sectional design, in which 101 adolescents age 15–16 years were recruited. The participants were assessed with the MABC-2 (eight tasks), the TMC (four tasks) and physical fitness measures (four tasks). Ninety-four participants completed all the test items (51% male). The correlation between the standard score of the MABC-2 and TMC total score was found to be moderate (r = −0.418). A weak correlation was found between MABC-2 and total score of physical fitness (r = 0.278), while the correlation between TMC and physical fitness was a little stronger (r = 0.361). However, when removing one measure from the TMC (the walking/running in slopes), the correlation was weak and not significant (r = 0.109). The results suggest that different test batteries can cause discrepancy in the results regarding correlation between motor competence and physical fitness in adolescents.

## 1. Introduction

An improved understanding of the relationship between factors that affect physical fitness (PF) in childhood and adolescence is valuable, as PF is a protective factor against health issues later in life [1,2,3]. Despite the lack of consensus on criterion measures of PF, it is accepted that health-related fitness measures must include muscular strength, flexibility, speed and endurance [4,5,6]. Motor competence (MC) is one of the possible predictive factors of PF level among youth, as MC is an essential interpreter of participation in physical activity (PA) during childhood and adolescence [7,8,9]. MC can be explained as a person’s ability to carry out different motor acts, including coordination of both fine (e.g., manual dexterity) and gross (e.g., static and dynamic balance) motor skills [10]. Earlier studies have revealed that children and adolescents with low MC tend to be less physically active, less likely to participate in sports and hold lower PF level related to their peers with high MC [11,12,13,14,15]. Stodden et al. [16] suggest that there is a reciprocal and developmentally dynamic relationship between MC and PA engagement in children and adolescents. Over time, this relationship is predicted to improve, that is, higher levels of MC will offer a better motor repertoire for participating in various physical activities. This makes the foundation for a positive spiral of engagement and thereby higher PF [16]. Previous studies on the long-term effects of motor skills intervention and daily PA during the compulsory school years revealed improvement in both motor skills and academic performance [17]. Additionally, high MC in adolescence serves as a protective factor against the decline in PA that is common in adolescence [18]. This evidence supports the development of motor skills and MC, as it influences children’s capacity to participate in PA and promotes a positive trajectory of health across the lifespan [19].

There is not yet a consensus on which constructs comprise MC, thus there is no “gold-standard” of measurement [20,21,22]. The choice of test battery for PF and MC depends on factors such as the suitability of the test for the age group, the purpose of the measurement and the facilities/environment where the assessment takes place [23,24]. Moreover, the test must be easy to administer, especially in educational settings. An evaluation of seven common movement skills assessment tools, namely Motoriktest für Vier- bis Sechsjährige Kinder (MOT 4–6) [25], Movement Assessment Battery for Children (MABC) [10], Peabody Developmental Motor Scales—Second Edition (PDMS-2) [26,27], Körperkoordinationstest für Kinder (KTK) [28], Test of Gross Motor Development-2 (TGMD-2) [29,30], Maastrichtse Motoriek Test (MMT) [31] and Bruininks–Oseretsky Test of Motor Proficiency (BOTMP; BOT-2) [32,33], revealed that they are all valuable and that their respective strengths and weaknesses depend on the intended purpose of the test [34]. Some MC tests have been developed to classify children with motor difficulties (e.g., MABC). These assessment tests have some limitations, as they are not equally sensitive at both ends of the scoring scale, leading to a ceiling effect [21]. Additionally, some MC tests are designed for certain age groups, and different tasks are administered to each age group, and this precludes longitudinal studies. Furthermore, comparison between countries is difficult due to a lack of standardisation, as different motor skill instruments are used in different countries [35]. The Test of Motor Competence (TMC) addresses this problem by measuring motor performance through the life span. The test’s tasks are sensitive at both ends of the distribution, allowing for above- and below-average scores. It also allows for longitudinal monitoring of MC because it is applicable to all ages [21].

Though there is no universal agreement on an optimal assessment of MC, most tests include tasks intended to measure both fine and gross motor skills. Regarding PF tests, consensus remains that the fitness components should include cardiovascular and muscular endurance, muscle strength, speed and flexibility [1,36]. These components all contribute to the development of children’s health and well-being [37,38]. There is a lack of evidence and research on the adolescent population concerning motor performance and its association with PF. Up to the present, few test batteries have been designed to measure MC in this age-group between 12–16 years, among these are MABC-2 [39], BOT-2 [32,33] and TMC [21]. The purpose of this study was twofold: first, to examine the correlation between scores on the MABC-2 and the TMC, and second, to interpret the correlation between PF measures and MC measures using these two different test batteries for MC.

## 2. Methods

### 2.1. Study Design and Participants

One hundred and one adolescents between 15 and 16 years of age were selected for this study. MC was assessed with the Movement Assessment Battery for Children-2 (second edition)(MABC-2) [39] and the Test of Motor Competence (TMC) [21]. Physical fitness was assessed using four tasks. Ninety-four adolescents (48 males and 46 females) completed all the test items. The complete sample was collected from two secondary schools in the capital city of Reykjavik, Iceland. The schools were randomly chosen, and the participants represented adolescents from a wide range of socio-economic backgrounds. The mean chronological age of the sample was 15.9 years (SD (Standard Deviation), 3.63 months), the range being from 15.4 to 16.3 years. The mean age for the girls was 15.8 years (SD, 3.62 months) and for the boys 15.9 years (SD, 3.63 months).

### 2.2. Study Measures

#### 2.2.1. Movement Assessment Battery for Children-2 (MABC-2)

The MABC-2 measures fine and gross motor coordination and is considered appropriate for ages 3–16 years [39]. The MABC-2 yields quantitative and qualitative assessment of MC in daily life for the child/adolescent. It is comprised of eight sub-tests, allocated into three groups: (1) manual dexterity (turning pegs, triangle with nuts and bolts, drawing trail), (2) ball skills (throwing at wall target, catching with one hand) and (3) static and dynamic balance (walking toe-to-heel backwards, zig-zag hopping, two-board balance). The raw scores for each of the eight items are summed and converted to a percentile rank. The test contains diverse tasks for children at different ages. For each age band, a given individual performance is referenced to a standardisation sample of an individual of the same age. Test–retest reliability of the total test score of 0.80, and an intra-rater reliability and inter-rater reliability of 0.79 indicates good reliability [39]. Regarding content validity, a panel of professionals judged the content of the MABC-2 to be typical of the motor domains as specified by the test developers [39]. In addition, the face validity was evaluated to be adequate (length of the test, the test appeal to children, levels of task and age-appropriateness) [39]. MABC-2 provides evidence that predictive validity and concurrent validity (between MABC-2, PDMS-2 and BOT-2) are moderate to high [40].

#### 2.2.2. Test of Motor Competence (TMC)

The TMC aims at quantifying MC across the life span [21]. TMC measures motor development, in both fine and gross motor skills. It is a product-based test, which is sensitive at both ends of the distribution. The test contains two fine motor tasks, based on manual dexterity, and two gross motor tasks based on dynamic balance. The performance measure was time to completion. Regarding internal consistency, all individual test item scores correlated positively with the total score (correlations ranging from 0.48 to 0.64) [21]. The Cronbach’s alpha value for the standardised test items was 0.79, which can be considered as acceptable [41]. Construct validity: the test has construct validity of 0.45 for 15–16-years-old (n = 101) for MABC-2 [21]. The MABC-2 is widely used in Nordic countries and Europe to measure children´s general MC and was therefore used to evaluate construct validity. As there is no “gold standard” to assess motor performance, there is always a question of what type of measurement one should compare with. Test–retest reliability: for the total score the TMC test–retest correlation coefficient was 0.87 [21].

Fine motor tasks: Placing bricks (PB) and building bricks (BB).

1. PB. Eighteen square-shaped Duplo™ bricks had to be placed on a Duplo™ board (3 × 6 bricks) as fast as possible. The adolescent was seated at a table and given a practice run before the testing. The bricks were positioned in horizontal rows of three on the side of the active hand and the board was held firmly with the other hand. Both hands were tested.
2. BB. Twelve square-shaped Duplo™ bricks were used to build a “tower” as fast as possible. The adolescent held one brick in one hand, and one brick in the other hand. At a signal, the adolescent assembled the bricks together one after the other, until all 12 were put together to form a tower. Arms were not allowed to rest on the table. The bricks had to be held in the air all the time. The tasks were conducted with the participants sitting comfortably at a table, and the time was stopped when the adolescent released contact with the last brick. Brick handling has been used extensively in previous test batteries [42].

Gross motor tasks: Heel-to-toe walking (HTW) and walking/running in slopes (W/R).

3. HTW. The adolescent walked down a straight line (4.5 m long) as fast as possible, placing heel against toe in each step.
4. W/R. On a signal, the adolescent walked/ran from the starting point, as fast as possible, in a figure of eight around two marked lines (1 m in width). Line 1 was 1 m from the starting point and Line 2 was 5.5 m from the starting point. If the adolescent started to go on the right side of Line 1, he/she had to go to the left side of Line 2, turn around, and go back on the right side of Line 2 and left side of Line 1, and over the starting point. The time was stopped (with a stop watch) when the adolescent arrived at the starting point. The participants freely chose which direction they walked/ran in.

#### 2.2.3. Testing of Physical Fitness

It is recommended that health-related fitness measures should include muscular strength, flexibility, speed and endurance [4,5,6]; therefore the following four tasks were selected: three tasks from the Test of Physical Fitness (strength, speed and endurance; tasks 1–3) [43] and one task from the Eurofit Fitness Test (flexibility; task 4) [44]. Tasks 1–3 had internal consistency (correlation with the total score) of 0.84 (standing broad jump); 0.88 (running 20 m); 0.65 (reduced Cooper test). The task had good test–retest reliability: standing broad jump intraclass correlation coefficient (ICC) 0.88; running 20 m ICC 0.71 and reduced Cooper test ICC 0.72. The task flexibility test from the Eurofit is found to be a reliable measure with an ICC of 0.91 in adolescents [44].

1. Standing broad jump. The adolescent stood with his/her feet parallel and shoulder width apart behind a starting line. Upon a signal, the adolescent swung his or her arms backward and forward and jumped with both feet simultaneously as far forward as possible. The test item score (the better of two attempts) was the distance (in centimetres) between the starting line and the landing position.
2. Running 20 m as fast as possible. The adolescent started in a standing position. On a signal, the participant ran as fast as possible toward the finish line. The test item score was the time in seconds needed to run the 20 m. If the adolescent made a procedural error, the performance was interrupted and the test item repeated.
3. Reduced Cooper test. The adolescent ran or walked around a marked rectangle measuring 9 × 18 m for 6 min. Both running and walking were allowed. The test item score was the distance reached in 6 min (in meters).
4. Sit and reach test. The adolescent sat on the floor with straight legs against a box and reached as far forward along the scale on the box pushing the ruler with extended fingertips. The adolescent should be able to hold the final position steady for 2–3 s without bouncing. The test box was 32 cm in height, with 45 cm wide top plate. The length of the top plate was 75 cm, the first 25 cm of which extended over the front edge of the box towards the subjects. The soles of the subject´s feet were placed against the front end of the box. The test item score was the length of the ruler push with hands on the box.

### 2.3. Procedure

The research was performed in accordance with the Declaration of Helsinki. The Icelandic National Bioethics Committee granted ethical approval (VSN09-009). The adolescents and parents were given written information about the study before data were gathered. Written consent was obtained from the participants and parents/guardian, and identification numbers were used to maintain data confidentiality.

The assessment of MC and PF occurred during school hours in a sports hall and was done in agreement with the test manuals. The adolescents were tested individually by assistants who had been trained in the test protocols. Each task item was explained and demonstrated before the participants started. Each participant was given verbal encouragement and support throughout the testing. If the participants made a procedural error, instructions and demonstrations were repeated and the participant made a new attempt. The adolescents were divided into two groups and the testing took 2 ½ hours for each participant. The participants wore clothing appropriate for PA and sport shoes throughout the testing.

### 2.4. Data Reduction and Analysis

All analyses were made in IBM SPSS statistics version 25 (IBM Corporation, New York, NY, USA). Task item scores for the four physical fitness tasks were converted into standardised scores (z-scores) using the mean of the whole sample for the age group. Z-scores were converted to ensure that the higher scores always indicated better performance than lower scores. The total test score per participant was calculated as the average z-score on all test items successfully performed by the same participant. The same procedure was used for the four tasks from TMC to obtain the total test score. A pilot study indicated that particularly one of the test items in TMC, walking/running in slopes, had a high correlation with total score PF. Analyses were therefore carried out both with the total score PF and the total score TMC, including all four test items and the total score TMC when the test item walking/running in slopes was excluded. Pearson’s correlation coefficient is the test statistic that measures the statistical relationship, or association, between two continuous variables. A high score indicated a high performance for the MABC-2, but a low score indicated high performance in TMC. For the physical fitness task, a high score indicated high performance in the standing long jump, 6-min running and the flexibility test. A low score indicated higher performance in the 20 m running task. With a sample size of 94, a correlation of 0.3 and significance level of 0.05, the power was estimated to be 0.84.

## 3. Results

The means and SD for age, measures of physical fitness and the measures of MABC-2 and TMC are presented in Table 1.

### 3.1. Association between MABC-2 Test and TMC Test

Correlations between the standard score of the MABC-2 and the total score of TMC were found to be significant and moderate (r = −0.418, *p* < 0.001). The correlations between standard score MABC-2 and the four measures of TMC were: PB (r = −0.401); BB (r = −0.300); HTW (r = −0.145); W/R (r = −0.168). The correlations between the standard score of the MABC-2 and the total score of TMC were found to be significant and moderate for both females (r = −0.398) and males (−0.457).

### 3.2. Association between PF and MC

The correlations between the standard score of the MABC-2, the total score of TMC (all four measures included and without walking/running in slopes), the four tasks of TMC and measures of PF by total score and gender are shown in Table 2. A significant but low correlation (r = 0.278) was found between MABC-2 and total score PF for the whole sample (female r = 0.358; male r = 0.327). A moderate significant correlation (r = −0.361) was found between TMC (all four measures included) and total score physical fitness for the whole sample (female r = −0.344; male r = −0.395). However, when removing one of the four measures within TMC (the walking/running on slopes task), the correlation was low and not significant (r = −0.109) (female r −0.197; male = −0.208).

## 4. Discussion

The study aimed to explore firstly, the correlation between two MC measures from the MABC-2 test and the TMC, and secondly, to interpret the correlations between PF measures and MC measures.

### 4.1. Motor Competence

The correlations between the standard score MABC-2 and total score of TMC were found to be significant and moderate (r = −0.418). This indicated that the total score of the two tests shared about one fifth of variance, suggesting that the two tests were measuring distinct aspects of MC. In relation to gender, there were only small differences (females: −0.398 and males: −0.457). These results are supported by other research studies indicating that there are distinctions between performance of various measurements of motor competence [20,45]. Logan et al. [46] compared the Test of Gross Motor Development—second edition (TGMD-2) and MABC-2 in children between five and seven years old and found only weak to moderate correlations between subscales and total performance on each assessment (r range between 0.27 to 0.52). Although most of the correlations were significant, the range of r^2^ values (between 0.07 to 0.27) demonstrated low levels of shared variance, indicating low practical significance [46]. In addition, in this sample, the mean performance on the TGMD-2 was significantly lower (17th percentile) than the MABC-2 (42nd percentile), supporting the argument that different aspects of motor competence were measured by the two test batteries. These results are extended and supported by Valentini et al. [24] in comparing the TGMD-2 and MABC-2 in children four to ten years old. Their study showed a weak correlation between total performance on the two tests (r = 0.23) and higher levels of better performance on the MABC-2 compared to the TGMD-2 across all ages. Ré et al. [45] compared the TGMD-2 and the KTK in children between five and 10 years and found only weak to moderate correlations (r range between 0.34 to 0.52) between tests across age, suggesting that the two tests are measuring distinct aspects of motor competence. Hence, various measurements of motor competence ought not to be used interchangeably [24,45,46]. To achieve a holistic measurement of an individual’s MC is challenging, because it is a variable composed of many different aspects [20].

Further analysis of the association among the motor competence test batteries indicated that the correlation between the standard score MABC-2 and the four measures of TMC were weak to moderate (ranging from −0.145 to −0.401). These findings were consistent with results in other studies that have found weak associations between different motor skills [47,48,49], and they may be explained by the specificity of motor skill learning [48,49,50,51,52,53,54]. The evidence of task specificity within the motor learning domain is a challenge when studying human movements. An example of this is the low correlation between total scores of different test batteries (between MABC and BOT (Bruininks-Oseretsky Test) the correlation is 0.53) [39]. It is interesting to note that there are higher correlations between the fine motor skills from TMC and the standard score of MABC-2 than between the gross motor skills from TMC and the standard score of MABC-2. Although difficult to interpret, it could be argued that the various test items within MC include different constraints in terms of speed, accuracy, sureness, coordination of the two hands, hand-eye coordination, hand-foot coordination and/or static/dynamic balance [39]. Even how the two different test batteries apply different procedures for scoring performance could influence the findings. Hence, MC is a variable composed of many different aspects and dimensions [20], and which set of skills that can provide a representative picture of the general MC is still unclear.

### 4.2. Motor Competence and Physical Fitness

The findings indicated a weak and significant correlation among MABC-2 and total score physical fitness (r = 0.278). The correlation between TMC (all four measures included) and total score physical fitness was stronger: r = −0.361. However, when removing one of the four measures within TMC (the walking/running on slopes task), the correlation was weakened and not significant (r = −0.109). This may indicate that the task walking/running on slopes also measured elements of fitness (muscle strength, speed/agility) in addition to motor competence. The weak relationship between the two factors may suggest that MC is not necessarily the most determinative factor for maintenance of PF in adolescents. In this age group, the amount of play-oriented activities decrease and participation in specific sports or physical activities that are less comprehensive in nature may become a more common choice [55,56]. For example, the performance of running may give restricted motor experiences in terms of manual dexterity or eye–hand coordination, but endorses merely the improvement of motor skills directly associated to the precise activity done, in this case dynamic balance. The intensity and duration of the physical activity could also be adequate to endorse PF (as muscle fitness and cardiorespiratory fitness). Thus, some physical activities could increase PF but not improve motor development [57]. On the contrary, other studies report moderate to strong significant correlations between MC and PF in children and adolescents [58,59]. The findings also reveal that the correlations among total score of PF and placing bricks, building bricks and heel-to toe walking in the TMC varied from r = 0.019 to r = −0.133, while the correlation to walking/running on slopes was r = −0.664. With respect to gender we saw that both males (−0.718) and females (−0.533) had a high correlation between walking/running on slopes and total score PF; however males had a higher correlation which may support the argument of muscle strength as a central component in this task.

Our findings in the study indicated the challenges with motor competence tests, when MC tasks include elements of strength and speed, components that are also included in PF measures. Different test items that could be difficult to distinguish from each other regarding what aspects of movement actually are being measured are not uncommon in studies [58,59,60]. This could cause co-dependency of the empirical data [61,62] For example, the task broad jump is a PF test measuring strength (anaerobic power), while the task single leg stationary hopping is operationalized as a measure of lower body gross MC [59]. Selection of the most appropriate test batteries requires careful comparison among the tests and analysis of their strength and weaknesses. An understanding of the specific objective of the test is also necessary for the interpretation of test results [23,63]. As stated by Utesch et al. [64], there is an overlap in content between measures of motor competence and physical fitness which need to be further investigated. From the literature, it seems useful to distinguish between the two theoretical constructs MC and PF [2,19]. However, it is possible to argue that this is not sufficiently reflected in how the variables are operationalized and measured. Finding a measurable set of skills that can provide a representative picture of the general MC of an individual across the life span poses a challenge. To expose the multidimensional construct of MC, test batteries that provide a more comprehensive way to measure children’s movement execution are suggested [20].

Few subjects participating (n = 94) in the study could be regarded as a limitation. Moreover, that the PF measure consisted of only four tasks can be a weakness, as the construct of PF consists of a variety of components such as body composition. On the other hand, the test items are mainly reflecting common activities, such as jumping and running which can support the ecological validity. As the construct validity of TMC is moderate (0.45), the results must be interpreted with caution. When considering the MABC-2, the overall reliability and validity of the instrument is under constant discussion [40]. However, it can be regarded as a strength that both MABC-2 and TMC are appropriate to measure MC in adolescents, as this is an understudied population regarding motor performance and its association with PF.

## 5. Conclusions

The results of this study reveal moderate significant correlations between the motor competences test batteries MABC-2 and TMC. The finding indicates weak correlation between MABC-2 and total score of PF and moderate significant correlations between TMC and PF. This study provides a compelling argument that different test batteries and interpretation of measurement can cause discrepancy in the results regarding correlation between MC and PF in adolescents. To achieve a holistic measurement of an individual’s MC is challenging, because it is a variable consisting of many different aspects [20]. One must find a measurable set of skills that can provide a representative picture of the general MC of an individual across the life span. Finding such a set of representative skills poses a significant challenge.

## Figures and Tables

**Table 1:** Descriptive statistics and motor competence (MC) and physical fitness (PF) scores.

Variable	Girls and Boys (n = 94)	Girls (n = 46)	Boys (n = 48)
	Mean (SD)	Mean (SD)	Mean (SD)
Age (years)	15.9 (3.63 months)	15.8 (3.62 months)	15.9 (3.63 months)
**Fitness Test**			
Standing broad jump (cm)	167.0 (42.77)	137.7 (29.53)	194.6 (34.09)
Running 20 m (s)	3.96 (0.46)	4.08 (0.35)	3.65 (0.45)
Reduced Cooper test (m)	1.124 (182)	1.057 (147)	1.185 (189)
Sit and reach test	30.09 (9.66)	35.1 (8.1)	25.53 (8.70)
**MABC-2**			
Standard Score MABC-2	9.16 (2.43)	9.44 (2.21)	8.91 (2.61)
Manual dexterity	7.04 (2.63)	7.90 (2.60)	6.24 (2.41)
Ball skills	10.35 (3.24)	9.35 (3.04)	11.35 (3.16)
Balance	11.66 (2.42)	12.16 (2.36)	11.20 (2.41)
**TMC**			
Placing bricks (s)	21.92 (2.25)	20.98 (1.87)	22.78 (2.23)
Building bricks (s)	12.87 (1.78)	12.42 (1.87)	13.27 (1.61)
Heel to toe walking	7.71 (1.68)	8.14 (1.58)	7.32 (1.68)
Walking/running in slopes	4.41 (0.65)	4.83 (0.57)	4.03 (0.46)

**Table 2:** Correlation (Pearson, two-tailed) between the standard score on the Movement Assessment Battery for Children version 2 (MABC-2), the total score for Test of Motor Competence (TMC) (with all four tasks), the total score for TMC (without walking/running on slopes), the four different tasks in TMC and physical fitness measure total score and gender.

Measures of Motor Competence	Total Score Physical Fitness Measure		
	Whole Sample (n = 94)	Girls (n = 46)	Boys (n = 48)
Standard score on the MABC-2	0.278 **	0.353 **	0.327 *
TMC (all four tasks)	−0.361 **	−0.344 *	−0.395 **
TMC (without walking/running on slopes)	−0.109	−0.197	−0.208
Placing bricks	0.019	−0.009	−0.274
Building bricks	−0.124	−0.317 *	−0.158
Heel to toe walking	−0.133	−0.082	−0.023
Walking/Running on slopes	−0.664 **	−0.533 **	−0.718 **

** correlation is significant at the 0.01 level (two-tailed), * correlation is significant at the 0.05 level (two-tailed).
